# A Case of Brain Abscess Caused by Medication-Related Osteonecrosis of the Jaw

**DOI:** 10.1155/2016/7038618

**Published:** 2016-02-02

**Authors:** Kenji Yamagata, Hiroki Nagai, Osamu Baba, Fumihiko Uchida, Naomi Kanno, Shogo Hasegawa, Toru Yanagawa, Hiroki Bukawa

**Affiliations:** Department of Oral and Maxillofacial Surgery, Institute of Clinical Medicine, Faculty of Medicine, University of Tsukuba, 1-1-1 Tennodai, Tsukuba, Ibaraki 305-8575, Japan

## Abstract

Reports of brain abscesses caused by medication-related osteonecrosis of the jaw (MRONJ) are very rare. We here present the case of a 76-year-old man with terminal-stage prostatic carcinoma and a brain abscess caused by MRONJ at the maxilla. The patient had been treated with zoledronic acid and denosumab for bone metastasis. For the brain abscess, an antibiotic regimen based on ceftriaxone and metronidazole and a sequestrectomy contributed to a successful outcome. In the case of maxillary MRONJ extending to the maxillary sinus, active resection of the infected bone should be considered to prevent the spread of the infection beyond the maxillary sinus, into the ethmoid sinus, and into the brain.

## 1. Introduction

A brain abscess is a rare, life-threatening infection. It appears as a localized area of suppuration within the brain. Although brain abscesses have historically been resistant to antimicrobial chemotherapy, recent advances in neuroscanning techniques such as CT and MR imaging and the introduction of more effective antibiotics have reduced the mortality rate to 0–25% [1, 2]. Brain abscesses spreading from the pericranial contiguous focus of the paranasal sinuses, the middle ear, or a dental infection are reported to account for 25–50% of the cases [2]. There are very few reports of a brain abscess secondary to odontogenic infection [3].

Osteonecrosis of the jaw is a common side effect of antiresorptive drugs [4], which are administered to cancer patients to treat bone metastasis, multiple myeloma, and osteoporosis. However, reports of a brain abscess resulting from medication-related osteonecrosis of the jaw (MRONJ) are very rare. We here describe the case of a brain abscess caused by a maxillary MRONJ that extended to the maxillary sinus and ethmoid sinus.

## 2. Case Report

**Figure 1:**
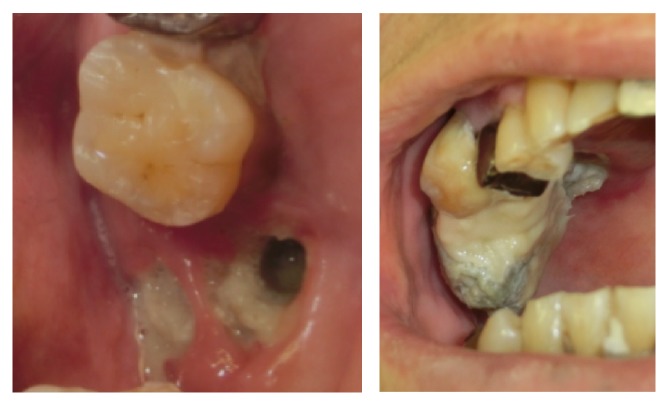
Intraoral examination.

**Figure 2:**
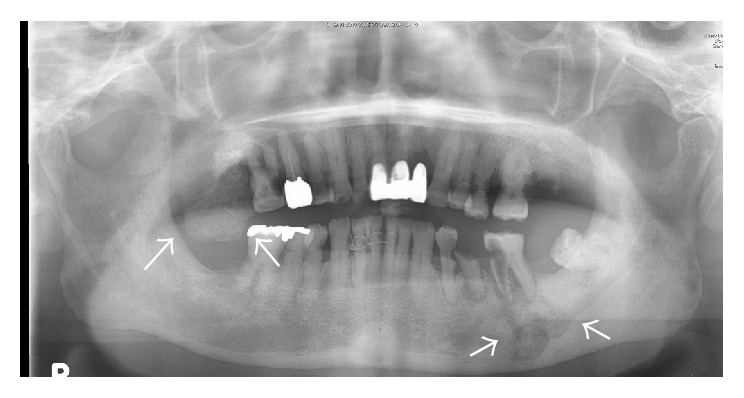
Preoperative panoramic radiograph.

**Figure 3:**
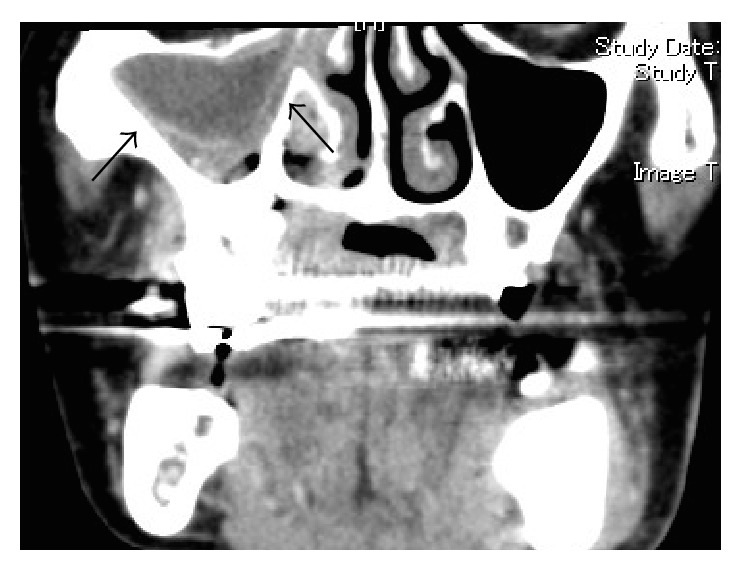
Preoperative CT revealing an abscess formation.

**Figure 4:**
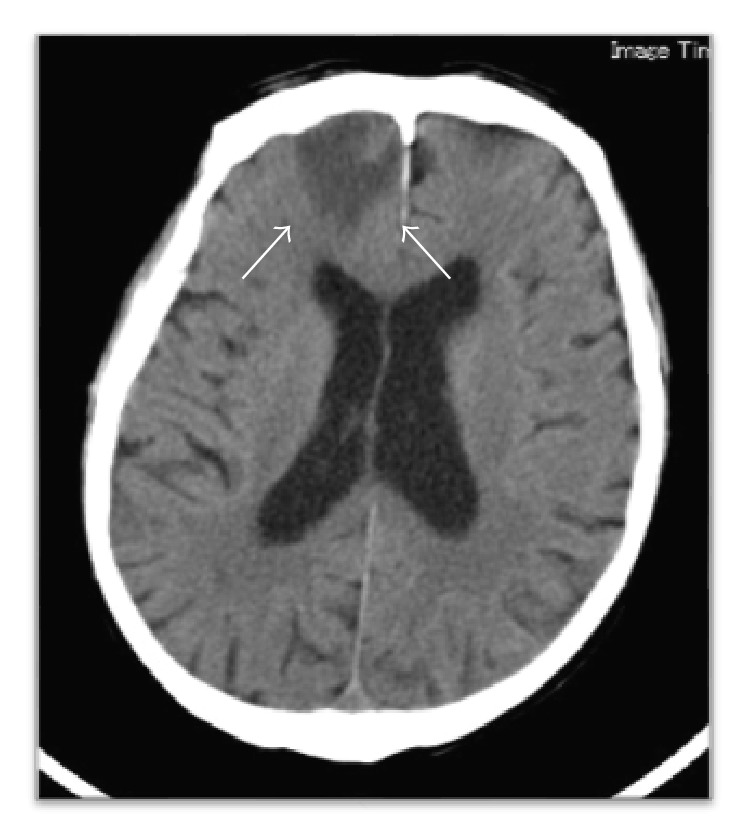
A CT showing a hypodense image in the right frontal lobe.

**Figure 5:**
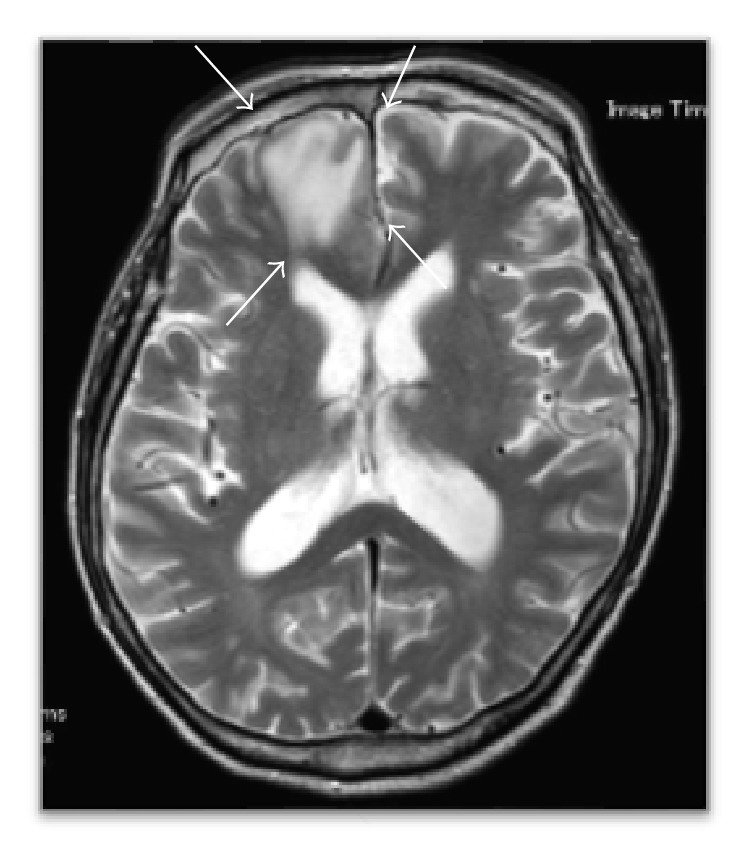
MR images (T2WI) showing hypointensity lesion in the right frontal lobe.

**Figure 6:**
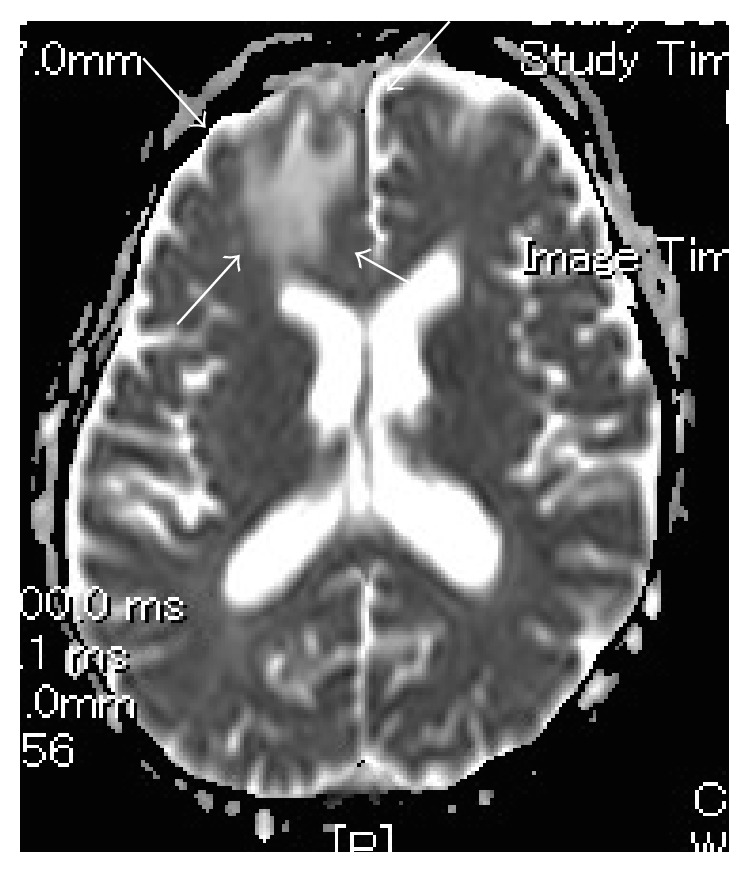
MR imaging (DWI) showing low apparent diffusion coefficient (ADC).

A 76-year-old man came to the Department of Oral and Maxillofacial Surgery, University of Tsukuba Hospital, with a three-month history of swelling of the left mandible. He had been diagnosed with stage IV prostatic carcinoma and multiple distant metastases and had been treated with zoledronic acid 4 mg for 22 times and denosumab 120 mg for 4 times. His face was symmetrical, with a fistula and pus discharge on the skin of the left mandible. The regional lymph nodes were normal, and there was no trismus. Examination of the oral cavity showed gingival swelling, redness, pus discharge, and loosening of teeth 35 and 36. Tooth 17 was lost naturally 2 months after the first visit, which revealed a sequestrum in the mouth; the sequestrum expanded over the next 8 months (Figure 1). A panoramic radiograph revealed separated sequestra of the right maxilla and the left mandible (Figure 2). Computed tomography (CT) revealed maxillary osteitis and sinusitis (Figure 3). An abscess had formed in the right maxillary sinus, and the patient complained of mild ophthalmalgia. Therefore, we performed a sequestrectomy of the right maxilla and drained the maxillary sinus under local anesthesia. The postsurgical course on day 0 was uneventful, and the patient was discharged from the hospital; however, he was found unconscious in his house on the day after surgery and was brought by ambulance to the emergency room (ER) at our hospital. The patient's consciousness level was 13 in the Glasgow Coma Scale (eye opening 4, verbal response 4, motor response 5), and he had left conjugate deviation and left hemiparesis. General convulsions occurred continuous to the left upper and lower limb convulsions. The convulsions were resolved by administering diazepam (5 mg) twice and fosphenytoin (900 mg) once. Laboratory results showed elevated WBC (10,300/*μ*L) and CRP (2.08 mg/dL). A brain CT revealed a hypodense lesion in the right frontal lobe (Figure 4). A T2-weighted MRI revealed a right frontal-lobe abscess and ethmoid sinusitis. The maxillary sinus was connected to the oral cavity, and the abscess in the maxillary sinus had disappeared (Figure 5). Diffusion-weighted imaging (DWI) MR imaging showed a low apparent diffusion coefficient (ADC) (Figure 6). A fall in the ADC in the fluid-filled lesion was highly suggestive of abscess.

The infection was suspected to have originated from the maxillary sequestrum and spread through the maxillary sinus, ethmoid sinus, and frontal sinus, and, by contiguity, to the frontal lobe. Although a bacterial culture of the maxillary sinus revealed only gram-positive and gram-negative bacilli and gram-positive cocci, these species were not found.

**Figure 7:**
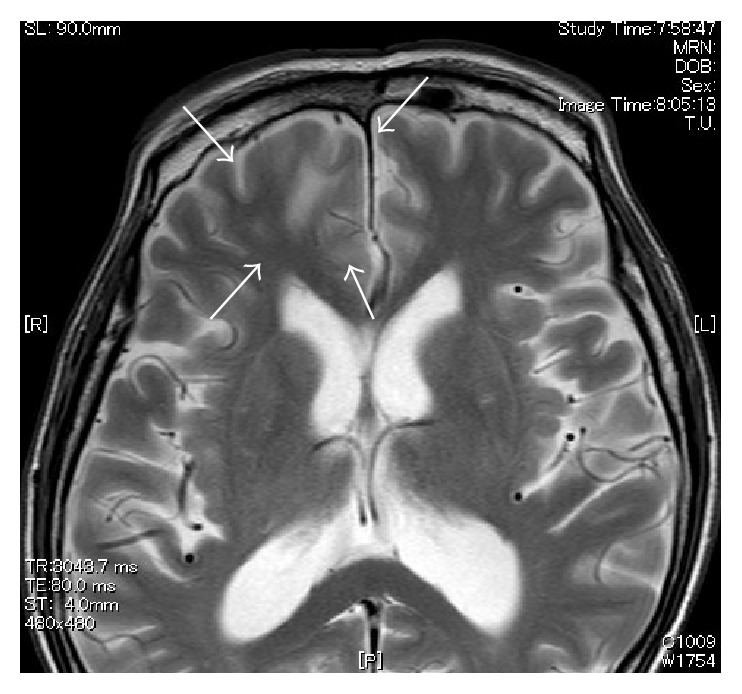
MR image (T2WI) obtained one month after beginning treatment.

The patient was treated with ceftriaxone 2 g q12h and metronidazole 500 mg q8h for 50 days, with valproate Na 800 mg for convulsions. The enhanced area of brain infection disappeared in follow-up MR images (Figure 7). This case was treated conservatively, without surgery, because the patient had terminal-stage cancer. The patient was discharged from the hospital after 2 months, without any left-limb paralysis.

## 3. Discussion

Brain abscess can originate exogenously, as when lesions are caused by skull trauma or surgery or, endogenously, from infections of continuous structures (e.g., otitis media sinusitis, mastoiditis, dental infections), from meningitis, or from the hematogenous spread of a remote infection (e.g., infective endocarditis, cyanotic congenital heart disease, pulmonary infections) [5]. A brain abscess caused by MRONJ is very rare.

The number of patients with MRONJ has grown recently. MRONJ is defined by the following characteristics: current or previous treatment with antiresorptive or antiangiogenic agents, exposed bone or bone that can be probed through an intraoral or extraoral fistula in the maxillofacial region that has persisted for more than 8 weeks, and no history of radiotherapy to the jaw or any obvious metastatic disease in the jaw [4].

A dental infection or ethmoid or frontal sinusitis that spreads to the brain generally causes a solitary brain abscess in the frontal lobe. Poor prognostic indicators include delayed diagnosis, a disease with rapid progression, coma, multiple lesions, intraventricular rupture, and fungal etiology, all of which are indicators seen in immunocompromised patients. Our patient was immunocompromised due to terminal prostate cancer. In the past, the overall mortality due to brain abscess was as high as 60%. Fortunately, new antibacterial approaches and new imaging technologies have decreased this mortality, and recent large case series have reported a mortality of 0%–25% [1, 2].

For a brain abscess originating from the paranasal sinuses, middle ear, or a dental infection, Arlotti et al. [5] recommend treatment with metronidazole 500 mg q8h and cefotaxime 2 g q6h or piperacillin/tazobactam 4.5 g q6h [5]. Accordingly, our patient was treated with ceftriaxone 2 g q12h and metronidazole 500 mg q8h for 50 days (ceftriaxone, like cefotaxime, is a third-generation cephalosporin). The pathogens related to this clinical scenario are the microaerophilic and anaerobic streptococci* Haemophilus* sp.,* Bacteroides* sp., and* Fusobacterium* sp. in the paranasal sinusitis and streptococci, gram-negative bacilli, and* B. fragilis* in the dental infection [2]. In the present case, a bacterial culture of the brain abscess revealed only gram-positive and gram-negative bacilli and gram-positive cocci.

The treatment of cerebral abscess includes the immediate administration of high-dose intravenous antibiotics, followed by surgical craniotomy and resection of the abscess cavity with the removal of possible septic foci [3, 6]. Our patient was immediately treated with high-dose intravenous antibiotics. However, we did not perform a craniotomy or resection of the frontal-lobe abscess because of the patient's poor general condition. The point of origin of the infection, the necrotic maxillary bone, was removed under anesthesia prior to the patient's loss of consciousness.

The clinical symptoms and signs are nonspecific for brain abscesses, which present differently depending on the origin, size, and location of the abscess [5]; the virulence of the infecting organisms; and the underlying systemic conditions. The most common symptoms are headache, nausea, vomiting, fever, focal neurological deficits, and an alteration of mental status [7]. Our patient presented postoperatively with loss of consciousness and general convulsions following convulsions of the left upper and lower limbs. The patient complained of mild ophthalmalgia before surgery; in retrospect, we suspected that the ophthalmalgia was a symptom of the brain abscess. The patient's early diagnosis, supported by imaging, was important for the successful outcome.

In conclusion, when maxillary MRONJ extends to the maxillary sinus, active resection of the infected bone should be considered to prevent the spread of the infection beyond the maxillary sinus, to the ethmoid sinus, and into the brain.
